# Precision medicine in Australia: indigenous health professionals are needed to improve equity for Aboriginal and Torres Strait Islanders

**DOI:** 10.1186/s12939-024-02202-7

**Published:** 2024-07-04

**Authors:** Dawn Alison Lewis, Tala Mitchell, Emma Kowal

**Affiliations:** 1 University of Adelaide, Adelaide, 5005 Australia; 2 Alfred Deakin Institute for Citizenship and Globalisation, Deakin University, Burwood Campus, 3125 Melbourne, Australia

## Abstract

Precision medicine, also known as “personalised medicine”, seeks to identify strategies in the prevention and treatment of disease informed by a patient’s genomic information. This allows a targeted approach to disease identification with the intention of reducing the burden of illness. Currently, both the emerging field of precision medicine and the established field of clinical genetics are highly reliant on genomic databases which are fraught with inbuilt biases, particularly from sample populations. The inequities of most concern here are those affecting Aboriginal and Torres Strait Islander (or Zenadth Kes) peoples of Australia (hereafter, respectfully, Indigenous Australians). It is with this perspective that the *Summer internship forINdigenous peoples inGenomics Australia* endeavours to support the development of culturally appropriate genomic research with Indigenous Australians. We argue here that Indigenous researchers are best placed to create the informed, culturally safe environment necessary for Indigenous Australians to participate in genomic research.

## Introduction

Inequities in health outcomes for Indigenous Australians are a result of ongoing and historical injustices [1–3]. Issues of access to health care, cultural and linguistic inclusion, physical accessibility, and racism all contribute to the broader issues of health outcomes for Indigenous Australians. Consequently, researchers are increasingly recognising the need to emphasise culturally competent research design [4, 5]. This means, for example, that research conducted in Indigenous communities must reflect Indigenous priorities and must provide benefits to that community [6]. However, what academics perceive as a benefit and what communities wish to receive or prioritise may not align and require further exploration. That is, are there secondary or even tertiary benefits that aren’t prioritised by researchers, but have more value for the community? Discussions such as these are integral to collaborative frameworks and a key reason the *Summer internship for Indigenous peoples in Genomics Australia* (SING Australia) holds an annual workshop. SING brings together Indigenous Elders, Indigenous community members with and without scientific backgrounds, health workers, and researchers from the scientific and humanities disciplines. The inaugural workshop was held in 2019 in response to the lack of Indigenous people engaged within genomic disciplines in Australia. As part of a global consortium, SING Australia is a sister-program to SING U.S.A, SING Canada, and SING Aotearoa. These are well established programs that have seen an increase in the development of Indigenous, Native and Maori people undertaking further education and training in genomics. SING Australia workshops are largely facilitated by Indigenous faculty who share their expertise, experience and leadership on topics related to DNA. SING Australia aims to enhance pathways and opportunities for Indigenous people through culturally safe discussions which address key issues in Indigenous Australian genomics. Through engaging Indigenous people from diverse backgrounds in discussions about genomics, SING Australia aims (1) to inspire Indigenous researchers to engage in the field of genomics research and (2) to inspire Indigenous health professionals to lead the implementation of precision medicine and other genomic health benefits for Indigenous people. Below, we explore the challenges faced in developing and delivering the SING Australia program, including managing the cultural sensitivities, discussing historic harm, and examining potential and actual benefits of precision medicine for Indigenous Australians.

### Genomics and culture

Having Indigenous populations (and other vulnerable groups) setting their own research goals and determining the benefit targets of research, more effectively mitigates potential harm from research practices [7]. Within genomic research, scholars have argued that understanding Indigenous worldviews is critical to ensuring that Indigenous people engage with, and benefit from, research [8]. For example, more than 15 years ago, Arbour L and Cook D [9] proposed the concept of “DNA on loan”, where Indigenous participants and communities are recognised as the owners of genomic samples that researchers temporarily “loan”. This concept was further developed by Indigenous geneticists with the note that gifting requires reciprocity or benefit sharing [10]. In broader, non-human applications, community inclusion in genomic research is important as Indigenous associations with land and community could be jeopardised by inappropriate, misunderstood, or miscommunicated genomic information [11]. Indigenous researchers are best placed to understand, and prioritise, community needs. Inclusive genomic spaces for Indigenous researchers have been shown to offer improved transparency, facilitation of improved dialogue between researchers and Indigenous communities, and the development of genomic research skills leading to greater participation and equity [12].

### Impacts of inequality in clinical services

Although genomic health care is currently a small part of the health system, inequalities in access still exist between Indigenous and non-indigenous Australians. As the role of precision medicine in the health care system increases, existing inequalities will grow without pre-emptive intervention. For Indigenous Australian families, clinical delays due to a lack of reference data are the leading cause of inequity in rare disease conditions [2]. It has been found that it can take up to five years for a rare disease diagnosis to be achieved for Indigenous Australians [13]. An accurate diagnosis improves clinical outcomes by ensuring medical care is appropriate and alleviating psychological, financial and social burdens for families [13]. Such life-changing diagnoses may be missed in minority populations that are not represented in reference data, leading directly to poorer clinical outcomes for these Indigenous families [14]. Clinical genetic services have a significant role to play in alleviating the challenges Indigenous peoples face in accessing genomic health care. In particular, the provision of support and resources for Indigenous people to undertake practitioner roles within such services, ideally servicing their own communities.

The Better Indigenous Genetics (BIG) project, led by the University of Melbourne, was the first systematic investigation of equity for Indigenous Australians who require genomic health care [15]. Focusing on both the health system and health service level, the project found that there was a willingness among Indigenous Australians to utilize clinical genomic services and that there is a higher incidence of genetic conditions amongst the population [15]. However, when genetic services are available, Indigenous Australians have lower rates of appointment scheduling and attendance [16]. In an indicative example, Aboriginal people of reproductive age (18–45 years) were 9.32 times less likely to be referred for a prenatal appointment within the Genetics Service of Western Australia than non-Indigenous people [15]. Of the Indigenous people who did access clinical genetic services, many reported that services displayed limited cultural understanding and inadequate assistance with travel and logistics, and experienced poor communication between genetic health practitioners [15, 17]. The 2021 census estimates that 582,000 (51%) Indigenous Australians live in regional and remote areas [18], and 167 different traditional languages are still spoken at home by 9.5% of the Indigenous population [19] [], indicating that attention to linguistic and logistic barriers, in addition to cultural barriers, is required for inclusive services. Current endeavours to address linguistic barriers in healthcare include the Western Australian Government department of Health’s support of the Lyfe Languages program [20]. Lyfe Languages is an Indigenous -led services which supports the high-fidelity translation of clinical terminology to local languages [21]. The expansion of similar services to other language groups requires the upskilling of health professionals with local language knowledge.

### Biobanking and clinical databases

Health inequalities between Indigenous and non-indigenous Australians remain significant [22, 23] and are largely explained by the prevalence of chronic diseases in Indigenous populations including diabetes, heart disease and respiratory disease [24–26]. It is likely that precision medicine will make increasing contributions to the management of chronic conditions. Precision medicine utilises genomic research to identify links between genetic variation and phenotype. The more robust a database of established genetic information, the more likely a genomic link will be identified, should it exist. As a result, these databases require the broadest range of source data; that is, genomic data that has come from many different populations around the world including Indigenous peoples. Unfortunately, these databases often consist of overrepresented populations, such as those of European ancestry, meaning that precision medicine tools based on those databases will be less accurate for non-European populations [26–28]. Indigenous Australians specifically are underrepresented in genomic and genetic research [29, 30] and clinical trials [26]. Indigenous populations around the world are likely to have unique genetic variants with unknown links to disease phenotypes [2, 27]. Recent work has exemplified the genetic stratification of Indigenous Australian populations pre-invastion [31] and post-invasion [32, 33] and reported extensive novel genetic variation across the continent. In order to ensure that precision medicine offers benefits to Indigenous peoples, it is imperative that references databased become more representative of world populations and more inclusive of Indigenous peoples.

Interviews with Indigenous Australians undertaken by the National Centre for Indigenous Genomics indicate an understanding and even enthusiasm for the use of biobanks in disease research (Hermes, et al. 2021). However, the inclusion of data from Indigenous populations in clinical databases is not straightforward. The misuse of Aboriginal clinical data has created mistrust between Indigenous Australians and health researchers [34, 35]. Indigenous Australians are now leading a new phase to decolonise the genomics space and enhance the workforce. The National Centre for Indigenous Genomics (NCIG) at the Australian National University has built unprecedented relationships with Indigenous communities, including returning biological samples to their communities of origin [35]. NCIG focuses on the informed consent not just of individuals, but of communities [Hermes et al., 2021). In this way, the Indigenous-majority board at the Centre recognises the non-individualistic nature of genomic data and respects community rights and values. Actively creating space for Indigenous people to support their communities is key in all aspects of Indigenous engagement, including genomics and precision medicine. The rapid developments in scientific and medical technologies have great potential for medical breakthroughs and quality of life improvements, but actual outcomes are uncertain until well tested. Given the nascent nature of technology used in precision medicine and therefore the uncertainty of outcomes and risks for novel technologies, Kendal E [36] questions whether informed consent is even possible in the context of precision medicine. This may be especially true for Indigenous communities wishing to consider the potential risks and reward of precision medicine from a communal perspective. An important step in addressing these challenges is to ensure that the scientific community includes Indigenous people with active ties to the communities they work in or with.

### Indigenous health workers

Indigenous groups have advocated over decades for self-determination and leadership in health [25]. By prioritising Indigenous healthcare workers - particularly in genomic and genetic medicine - culturally appropriate, informed interpretation of individual genetic information is possible. The National Aboriginal Torres Strait Islander Health Workforce Strategic Framework [37] has identified six priority areas to support the health workforce. These include strengthened recruitment and retention of Indigenous health professionals, building on workforce capacity, promotion of the benefits of culturally safe workplaces, improving the number of students with health qualifications, and supporting workforce policy and planning [37]. Within this framework, there is no mention of the need to prioritize practitioners in clinical genetics and precision medicine. It would be extremely beneficial if a dedicated framework for workforce capacity in genomics was developed and underpinned by the priorities and values of Indigenous Australian communities.

Unfortunately, there are only a handful of Aboriginal geneticists in Australia, and at present, there are only two Aboriginal or Torres Strait Islander genetic counsellors and no clinical geneticists. Indigenous clinicians would be best placed to ensure the information disseminated about precision medicine is linguistically and culturally accessible or identify when this is not the case.

## Conclusion

International literature has highlighted the importance of addressing racial and ethnic workforce disparities in increasing access to genetic services for underserved communities [38]. A strengthened Indigenous Australian genomics workforce will allow for a greater voice and participation in the health system [39]. Indigenous genetic clinicians, research personnel and scientific lab technicians are needed to provide a bridge between research, its outcomes and benefits, and community needs, wants and concerns. Workshops and programs such as SING Australia allow professionals to form cross-disciplinary networks that support their own careers. SING Australia brings together Indigenous academics, professionals, students, and community members to ensure concerns are actively heard within academic circles and research is explained appropriately to community decision-makers, while building capacity for students to continue into genomic research. Through inspiring the next generation of Indigenous researchers and clinicians, the future health benefits of genomics can be equitably shared and Indigenous communities can be empowered to lead their own research and determine their own benefits.

## Data Availability

N/A.
